# Global Burden of Drug‐Induced Anaphylaxis Associated With 33 Classes of Antibiotics (1968–2024): A Pharmacovigilance Analysis

**DOI:** 10.1111/cea.70121

**Published:** 2025-07-29

**Authors:** Jaehyeong Cho, Jeongseon Oh, Jaeyu Park, Hyesu Jo, Tae Hyeon Kim, Hyunjee Kim, Yesol Yim, Seoyoung Park, Kyeongeun Kim, Ho Geol Woo, Yerin Hwang, Michael Miligkos, Dong Keon Yon, Nikolaos G. Papadopoulos

**Affiliations:** ^1^ Center for Digital Health Medical Science Research Institute, Kyung Hee University College of Medicine Seoul South Korea; ^2^ Department of Medicine CHA University School of Medicine Seongnam South Korea; ^3^ Department of Precision Medicine Kyung Hee University College of Medicine Seoul South Korea; ^4^ Department of Regulatory Science Kyung Hee University Seoul South Korea; ^5^ Department of Medicine Kyung Hee University College of Medicine Seoul South Korea; ^6^ Department of Neurology Kyung Hee University Medical Center, Kyung Hee University College of Medicine Seoul South Korea; ^7^ Allergy Department, 2nd Pediatric Clinic National and Kapodistrian University of Athens Athens Greece; ^8^ Department of Pediatrics Kyung Hee University Medical Center, Kyung Hee University College of Medicine Seoul South Korea; ^9^ Lydia Becker Institute, University of Manchester Manchester UK

**Keywords:** allergy, anaphylaxis, antibiotics, pharmacovigilance

## Abstract

**Background:**

Despite antibiotic‐induced anaphylaxis being a severe allergic reaction requiring immediate care, large‐scale studies examining all antibiotic subtypes remain limited. This study addresses this gap by analysing 33 antibiotic classes, along with epidemiological and regional variations.

**Methods:**

This study utilised the world's largest pharmacovigilance database, with over 35 million individual case safety reports from 140 countries. The study employed a two‐step approach: first, the antibiotics were grouped into 10 categories according to their third‐level ATC codes and their frequencies were collectively analysed. These categories comprised tetracyclines (J01A), amphenicols (J01B), beta‐lactam antibacterial penicillins (J01C), other beta‐lactam antibacterials (J01D), sulfonamides and trimethoprim (J01E), macrolides, lincosamides and streptogramins (J01F), aminoglycoside antibacterials (J01G), quinolone antibacterials (J01M), combinations of antibacterials (J01R) and other antibacterials (J01X). Second, a more detailed analysis was performed at the fourth level of the ATC codes for the antibiotics categorised at the third level, focusing on 33 individual antibiotics. For statistical analysis, disproportionality metrics, including the information component (IC) with IC_025_ and reporting odds ratio (ROR) with 95% CI, were used to classify and analyse the risk of anaphylaxis related to these drugs.

**Results:**

A total of 144,820 reports were identified as antibiotic‐induced anaphylaxis. All antibiotics showed significant signal detection for anaphylaxis (ROR, 20.50 [95% CI, 20.37–20.63]; IC, 3.77 [IC_025_, 3.76]) across all age groups and sexes. The following three antibiotics took the most proportion of the reports: penicillins (39,696/144,820 [27.4%]; ROR, 18.82 [95% CI, 18.62–19.01]; IC, 4.04 [IC_025_, 4.02]), other beta‐lactam antibiotics (63,644/144,820 [43.9%]; 27.59 [27.35–27.83]; 4.48 [4.46]) and quinolones (20,303/144,820 [14.0%]; 13.40 [13.21–13.60]; 3.63 [3.61]). The median time‐to‐onset was 1 day (interquantile range, 1–1), with most r recovered (96.09%) and the fatality rate accounting for 1.23%.

**Conclusion:**

Although our findings do not permit causal inference, the analysis highlights the need for standardised grading systems, patient‐specific risk factors and long‐term outcome studies to improve prevention and management.

## Graphical abstract

Beta‐lactam antibacterials, including penicillins and other beta‐lactam antibacterials showed the highest signal strength and reporting frequency. These findings underscore the importance of evaluating the long‐term outcomes of antibiotic‐induced anaphylaxis to improve prevention and management strategies.

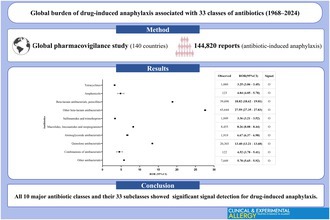

**Abbreviations**
ADRs: adverse drug reactions
ATC: anatomical therapeutic chemical
CI: confidence interval
IC: information component
ICSRs: individual case safety reports
ROR: reporting odds ratio
TTO: time‐to‐onset

**Summary**

- All antibiotics showed significant anaphylaxis signals, with lower IC values in younger patients.
- Penicillins, beta‐lactams and quinolones accounted for approximately 85.4% of anaphylaxis reports.
- Median TTO was 1 day; 96.1% recovered, and only 0.12% resulted in death.

## 1 Introduction

Anaphylaxis is a severe and acute allergic reaction, with an incidence of 50–112 cases per 100,000 person‐years and an estimated lifetime prevalence of 0.3%–5.1% [1]. Despite the low mortality rate, hospitalisations for drug‐induced anaphylaxis have increased in recent years [2], with estimates of approximately 0.05–0.51 per million person‐years for drug‐induced cases [3].

Following the well‐established finding that antibiotics are the primary cause of drug‐induced anaphylaxis, many studies have explored this relationship. However, most of these investigations were constrained by geographic and ethical factors [4], limiting their ability to conduct large‐scale, diverse population‐based analyses in real‐world settings. Furthermore, to our knowledge, most previous studies have focused on particular antibiotic subtypes rather than examining all possible subtypes [5]. In addition, since anaphylaxis is often categorised under the broader domain of allergic reactions, much of the existing research has focused on overall allergic reactions in general rather than on anaphylaxis specifically [6].

To address these gaps, our study utilized the global pharmacovigilance database to systematically examine anaphylaxis adverse events and its detected signal with various antibiotic subtypes. By leveraging this extensive and internationally representative dataset, we aim to evaluate global reporting patterns of antibiotics and investigate potential demographic and regional variations. This comprehensive approach enhances our understanding of antibiotics linked to drug‐induced anaphylaxis and is expected to contribute to improved pharmacovigilance and patient safety strategies.

## 2 Methods

### 2.1 Data Sources

The international pharmacovigilance database has collected spontaneous reports of adverse drug reactions (ADRs) from post‐marketing use since 1968, with some countries also submitting data from pharmaceutical companies during clinical trials. It now contains over 35 million individual case safety reports (ICSRs) from more than 140 countries, having grown exponentially over time and incorporating most of the data from other major databases, including those of the European Union, Japan and the United States [7, 8]. ADRs have been reported by health professionals, pharmaceutical companies and consumers to national pharmacovigilance authorities [9]. The present research was conducted using fully anonymised patient data following the institutional review board of Kyung Hee University. As the dataset does not contain any personally identifiable information, the requirement for informed consent was waived for this study.

### 2.2 Selection of Cases

We included all reports from 1968 through June 30, 2024, associated with the following preferred terms related to anaphylaxis: ‘Anaphylactic reaction’, ‘Anaphylactic shock’, ‘Anaphylactoid reaction’ and ‘Anaphylactoid shock’, using version 26.0 of the Medical Dictionary for Drug Regulatory Activities (MedDRA) [10]. Only drugs labelled as suspected were analysed (Table S1). In addition, the International Classification of Diseases, Tenth Revision, is the classification system most widely used by the global allergy community, but it is not considered appropriate for clinical practice [11]. Consequently, employing MedDRA codes to identify anaphylaxis‐related adverse events may hold greater clinical significance.

To ensure consistency and reliability, we restricted our analysis to antibiotics with Anatomical Therapeutic Chemical (ATC) codes beginning with ‘J01’. A separate categorisation analysis was then conducted to examine the frequency of anaphylaxis‐related reports with specific antibiotics. The study employed a two‐step approach: first, the antibiotics were grouped into 10 categories according to their third‐level ATC codes, and their frequencies were collectively analysed. These categories comprised tetracyclines (J01A), amphenicols (J01B), beta‐lactam antibacterial penicillins (J01C), other beta‐lactam antibacterials (J01D), sulfonamides and trimethoprim (J01E), macrolides, lincosamides and streptogramins (J01F), aminoglycoside antibacterials (J01G), quinolone antibacterials (J01M), combinations of antibacterials (J01R) and other antibacterials (J01X) [12]. Second, a more detailed analysis was performed at the fourth level of the ATC codes for the antibiotics categorised at the third level, focusing on 33 individual antibiotics [12]. The categorisation into beta‐lactam antibacterial penicillins and other beta‐lactam antibacterials is determined by the inclusion or exclusion of penicillins, respectively [13]. The other antibacterials (J01J) category includes drugs outside the major classes such as penicillins, cephalosporins and tetracyclines, encompassing those classified under the fourth ATC level, including glycopeptide antibacterials, polymyxins, steroid antibacterials, imidazole derivatives and nitrofuran derivatives [14].

### 2.3 Data Collection

For this study, data were stratified by region (Africa, the Americas, Southeast Asia, Europe, the Eastern Mediterranean and the Western Pacific), reporting period (1968–1979, 1980–1989, 1990–1999, 2000–2009, 2010–2019 and 2020–2024), reporter category (healthcare professionals, non‐healthcare professionals or unknown), sex (male and female), age group (0–17, 18–44, 45–64, 65–74 and ≥ 75 years), time‐to‐onset (TTO) and fatal outcomes (recovered, not recovered, death, or unknown), as well as detailed information on adverse drug reactions [15]. Furthermore, TTO was calculated in days from the date of initiation of treatment to the date of onset of the effect (TTO = effect onset date—drug start date).

### 2.4 Statistical Analysis

A disproportional analysis using data from the global pharmacovigilance database was performed employing two measures: the reporting odds ratio (ROR) and the information component (IC). The ROR, which approximates the odds ratio used in case–control studies, was used to assess the strength of disproportionality by comparing reports linked to anaphylaxis with all other ICSRs [16]. Statistical significance was defined as the lower bound of the 95% confidence interval (CI) exceeding 1.00. The IC, a Bayesian disproportionality measure used for signal detection, enhances the identification of rare ADRs by accounting for under‐reporting and variability in spontaneous reporting systems, providing a more reliable assessment of drug‐event associations than traditional disproportionality methods [16, 17, 18]. In keeping with standard IC analysis, an event was considered disproportionately over‐reported if the lower endpoint of the 95% credibility interval for the IC was positive (IC_0.25_ > 0.00). All statistical analyses were performed using SAS software (version 9.4; SAS Institute, Cary, NC, USA).

## 3 Results

### 3.1 Overall Analysis

**FIGURE 1:**
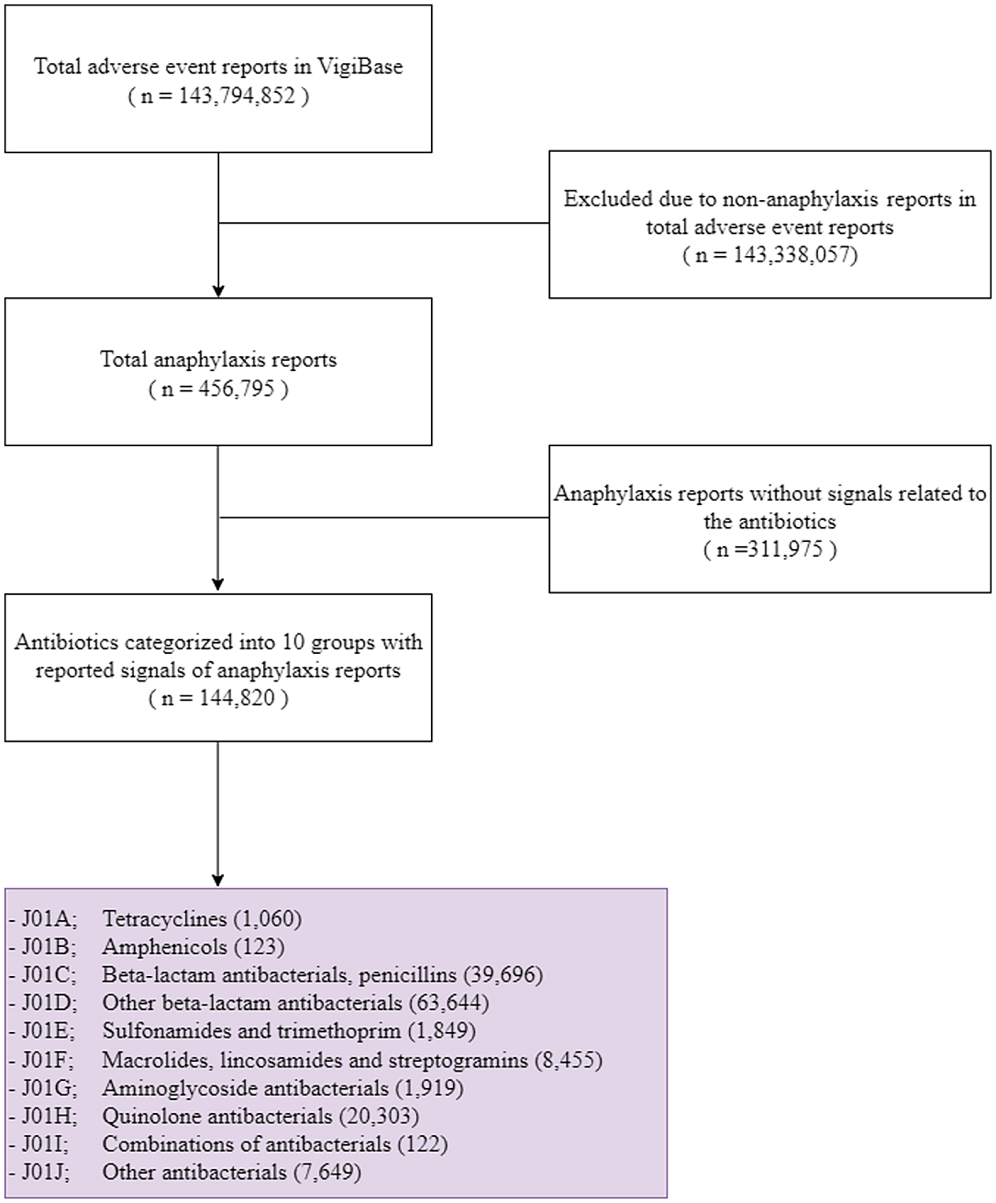
Flow chart illustrates the selection process for antibiotics‐associated anaphylaxis events.

**FIGURE 2:**
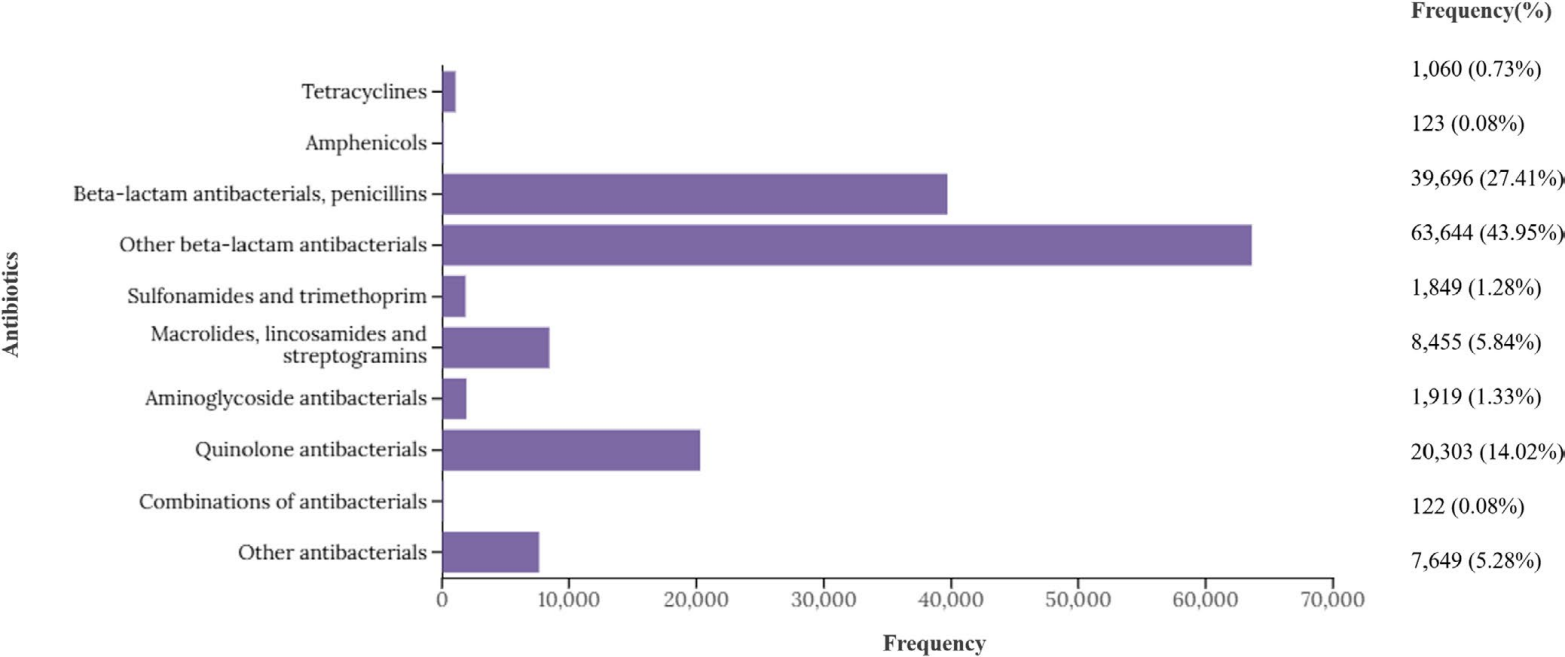
Distribution of drug‐induced anaphylaxis reports across 10 antibiotic categories classified at the ATCcode 4th levels.

Among the over 35 million ICSRs reported in the database, 144,820 reports were identified (Figure 1). The Western Pacific region accounted for the highest proportion of reports (71.74%), followed by Europe (13.74%), Southeast Asia (7.52%) and America (6.00%) (Table 1). More than half of the reports were observed in the working‐age group (18–64 years; 60.97%) compared to the young age group (1–17 years; 10.57%) and the old age group (≥ 75 years; 9.8%). In addition, a higher proportion of reports was observed in females (53.96%) compared to males (44.76%). As shown in Figure 2, three antibiotic classes, including penicillins (27.41%), other beta‐lactam antibiotics excluding penicillins (43.95%) and quinolones (14.02%), accounted for 85.38% of overall drug‐induced anaphylaxis reports. Among them, antibiotics targeting beta‐lactam ring inhibition comprised the largest proportion among the 10 analysed categories, representing 71.36% of total reports. Cumulative data show a steady increase in overall report counts since the database was established in 1968 (Figure S1). Initially, penicillins accounted for approximately 80% of reports, but over time, the proportion of other beta‐lactam antibiotics and quinolones has steadily increased, making them the top three antibiotic classes that showed significant signal detection for drug‐induced anaphylaxis.

**TABLE 1:** Baseline characteristics of reports on antibiotic‐induced anaphylaxis events.

Variables		Number (%)
Region reporting	African region	352 (0.24)
	Region of the Americas	8694 (6.00)
	Southeast Asia region	10,896 (7.52)
	European Region	19,893 (13.74)
	Eastern Mediterranean region	1093 (0.75)
	Western Pacific region	103,892 (71.74)
Reporting year	1968–1979	415 (0.29)
	1980–1989	1571 (1.08)
	1990–1999	4048 (2.80)
	2000–2009	6781 (4.68)
	2010–2019	69,086 (47.70ㅍㅑ햐ㅠ)
	2020–2024	62,919 (43.45)
Reporter qualification	Health professional	140,434 (96.97)
	Non‐health professional	4386 (3.03)
Sex	Male	64,823 (44.76)
	Female	78,144 (53.96)
	Unknown	1853 (1.28)
Age, years	1–17 year	15,313 (10.57)
	18–44 years	42,033 (29.02)
	44–64 years	46,273 (31.95)
	65–74 years	20,935 (14.46)
	≥ 75 years	14,191 (9.80)
	Unknown	6075 (4.19)
TTO, days	Median days (IQR)	1 (1–1)
Fatal outcomes	Recovered	129,702 (89.56)
	Not recovered	3435 (2.37)
	Fatal	1672 (1.15)
	Death	169 (0.12)
	Unknown	9842 (6.80)
Single drug suspected		144,820 (100.00)

Abbreviations: IQR, interquartile range; TTO, time‐to‐onset.

### 3.2 Disproportionality and Subgroup Analysis of Drug‐Induced Anaphylaxis

**FIGURE 3:**
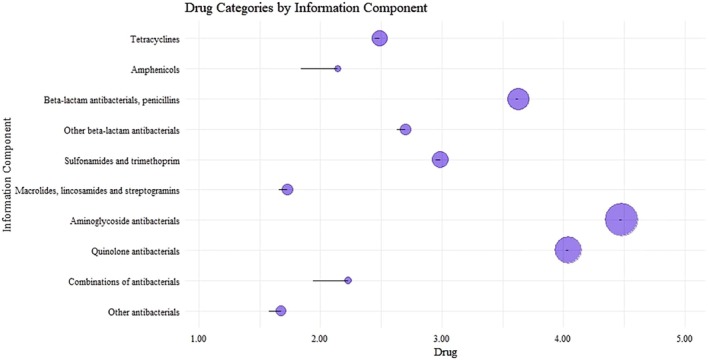
Analysis of subgroup IC_0.25_ values for different drugs: Anaphylaxis adverse events disproportionality. IC, information component.

**FIGURE 4:**
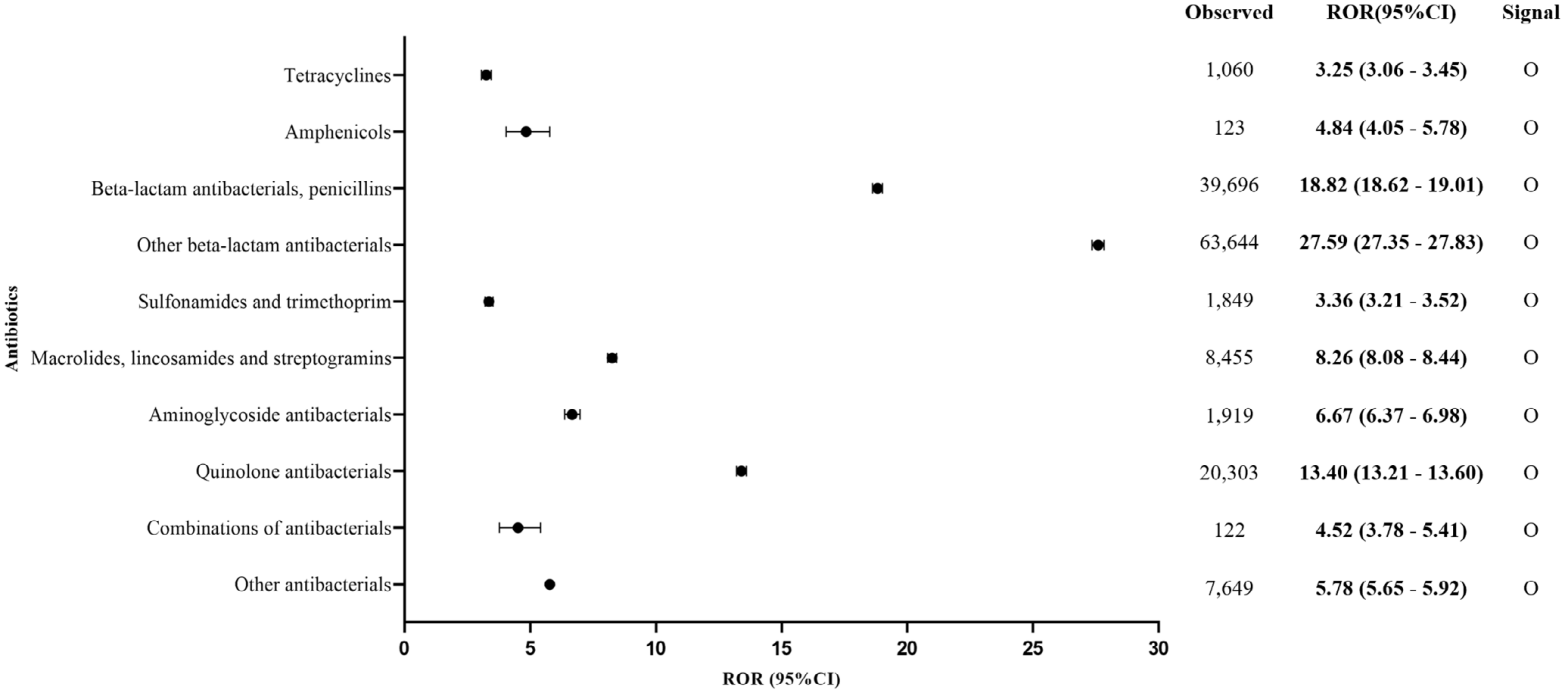
Analysis of subgroup ROR values for different drugs: Drug‐induced anaphylaxis disproportionality. CI, confidence interval; ROR, reporting odds ratio.

The overall signal between all antibiotic subtypes and drug‐induced anaphylaxis was significant (ROR, 20.50 [95% CI, 20.37–20.63]; IC, 3.77 [IC_0.25_, 3.76]) in both males (19.70 [19.51–19.90]; 3.65 [3.64]) and females (20.93 [20.75–21.11]; 3.84 [3.83]) (Table S2). Consistent with the lower number of reports in children and adolescents, the statistical value for anaphylaxis risk was also lower in this group (1–17 years: IC, 3.06 [IC_0.25_, 3.03]) compared to other age groups (18–44 years: 3.37 [3.35]; 45–64 years: 3.77 [3.76]; 65–74 years: 3.80 [3.78]; ≥ 75 years: 3.61 [3.58]). Notably, all 10 major antibiotic classes and their 33 subclasses showed significant signal detection for drug‐induced anaphylaxis. Specifically, three antibiotic classes with higher numbers of reports exhibited higher ROR and IC values: penicillins (39,696/144,820; ROR, 18.82 [95% CI, 18.62–19.01]; IC, 4.04 [IC_0.25_, 4.02]), other beta‐lactam antibiotics (63,644/144,820; 27.59 [27.35–27.83]; 4.48 [4.46]) and quinolones (20,303/144,820; 13.40 [13.21–13.60]; 3.63 [3.61]). In contrast, antibiotic classes with fewer reports showed lower ROR and IC values, including tetracyclines (1060/144,820; ROR, 3.25 [95% CI, 3.06–3.45]; IC, 1.68 [IC_0.25_, 1.58]), amphenicols (123/144,820; 4.84 [4.05–5.78]; 2.23 [1.94]), sulfonamides and trimethoprim (1849/144,820; 3.36 [3.21–3.52]; 1.73 [1.66]), macrolides, lincosamides and streptogramins (8455/144,820; 8.26 [8.08–8.44]; 2.99 [2.95]) and aminoglycosides (1919/144,820; 6.67 [6.37–6.98]; 2.70 [2.63]). In addition, two less common antibiotic categories also showed significant signal detection with drug‐induced anaphylaxis: combinations of antibiotics (122/144,820; 4.52 [3.78–5.41]; 2.14 [1.84]) and the group comprising glycopeptide antibiotics, polymyxins, steroids, imidazole derivatives and nitrofuran derivatives (7649/144,820; 5.78 [5.65–5.92]; 2.49 [2.45]) (Figures 3 and 4).

### 3.3 Clinical Features of Drug‐Induced Anaphylaxis in Reports

**FIGURE 5:**
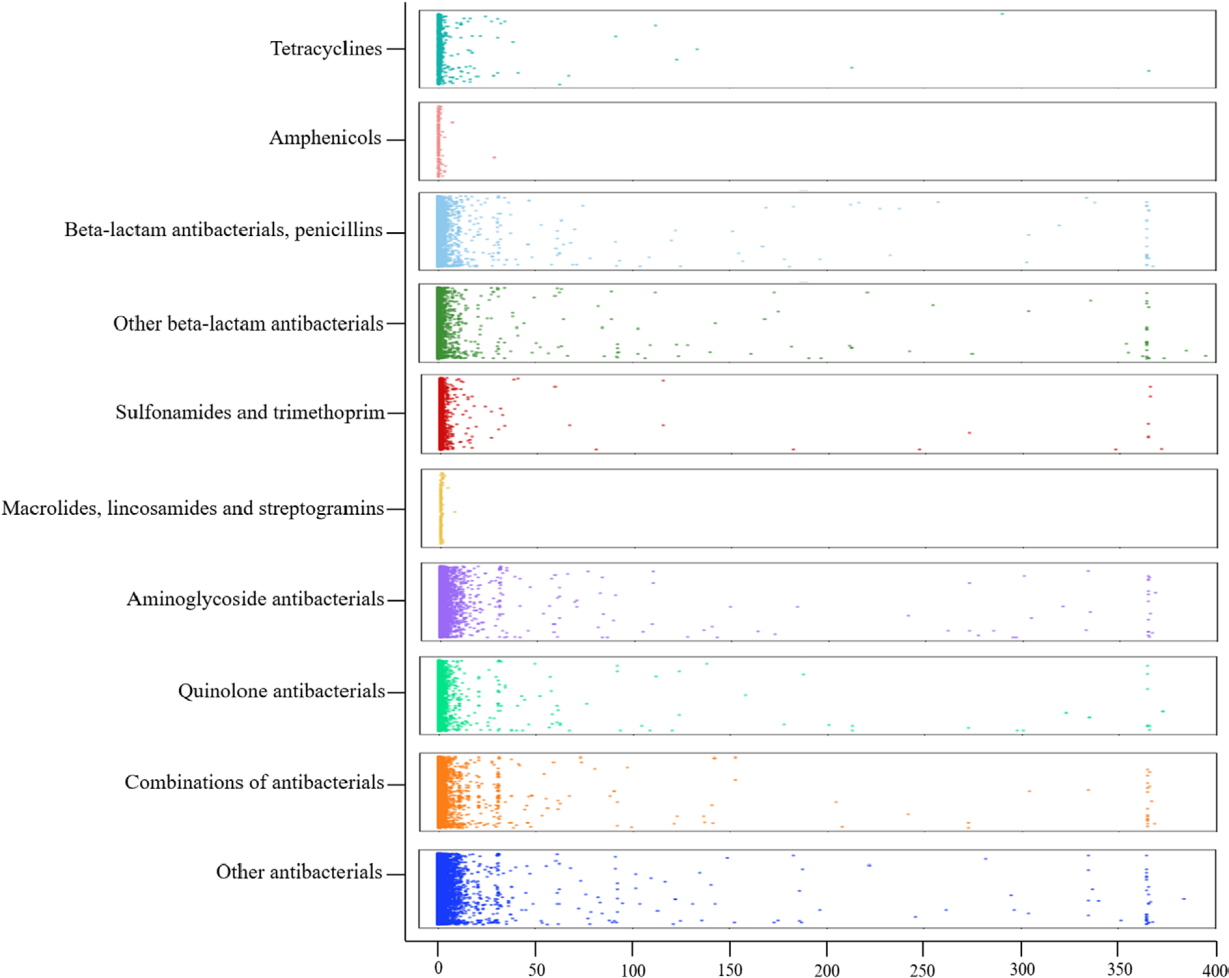
Timeline of drug‐induced adverse reaction onset following administration of 10 antibiotic categories classified at the ATC code 4th levels.

The median days of TTO for drug‐induced anaphylaxis was one day across all 10 antibiotic classes (Figure 5). Excluding reports with unknown outcomes, a remarkably high proportion of individuals (96.09%) recovered from the anaphylaxis, while fatalities accounted for 1.23% of reports.

## 4 Discussion

### 4.1 Key Findings

This study comprehensively analysed drug‐induced anaphylaxis, emphasising its signal detection for various antibiotics used worldwide. Most of the 144,820 reports of drug‐induced anaphylaxis were reported in the Western Pacific region, followed by Europe, Southeast Asia and America. All antibiotics included in the analysis detected a significant signal for drug‐induced anaphylaxis, with consistent findings across both males and females. However, the younger age group (1–17 years) had fewer reports and a lower IC value compared to older age groups. Specifically, penicillins, other beta‐lactam antibiotics and quinolones accounted for the largest proportion of drug‐induced anaphylaxis reports, comprising approximately 85.38% of the total reports. Since the establishment of the global pharmacovigilance database in 1968, the cumulative number of reports has steadily increased. In line with reporting trends, these antibiotics also represented the highest ROR and IC values. The median TTO for drug‐induced anaphylaxis was one day, and a notably high percentage of individuals (96.09%) recovered from the condition, with an extremely low death rate (0.12%).

### 4.2 Comparison of Previous Studies

Since antibiotics are the most common cause of life‐threatening immune‐mediated drug‐induced anaphylaxis, numerous studies have attempted to investigate their association with allergic reactions [4, 5, 6, 19, 20]. However, most of these studies are limited, as they primarily focus on specific antibiotic classes [4, 5, 6, 19], a small number of cases [4, 5, 19], or restricted patient populations [4, 5, 19, 20] when assessing anaphylaxis risk. Furthermore, most previous studies have focused on overall allergic reactions, including anaphylaxis, rather than specifically examining drug‐induced anaphylaxis [4, 6]. Additionally, studies using other pharmacovigilance databases face limitations in periods and coverage of data collection, making it difficult to fully capture the global landscape of antibiotics‐induced anaphylaxis [21].

However, our analysis leveraged the pharmacovigilance database, a large global dataset encompassing all 33 antibiotic classes classified under the ATC system [12]. Additionally, although ICD‐10 is widely used in the global community, it is not ideal for clinical practice [11]; therefore, using MedDRA codes to identify anaphylaxis‐related adverse events may provide greater clinical relevance, focusing solely on anaphylaxis [22, 23, 24]. With these efforts, our study found that beta‐lactam‐related antibiotics accounted for the largest proportion of reports, consistent with previous studies highlighting the widespread use of beta‐lactam antibiotics globally [25]. Additionally, consistent with the well‐established understanding that drug‐induced anaphylaxis is more prevalent in adults than in children [26], our results also showed fewer reports and lower statistical values in children and adolescents.

### 4.3 Underlying Plausible Mechanisms

A recent study shows that both IgE‐mediated and non‐IgE‐mediated pathways can contribute to immunologic hypersensitivity reactions [27]. Additionally, non‐beta‐lactam antibiotics, including macrolides and lincosamides, may induce anaphylaxis through alternative mechanisms, such as T‐cell activation or the release of cytokines like interleukin (IL)‐4 and IL‐13, further amplifying immune responses [28]. Furthermore, regional variations in reports may be influenced by differences in antibiotic prescribing patterns, healthcare accessibility and the effectiveness of pharmacovigilance systems. The Western Pacific region, which includes countries such as China, Japan and South Korea, has high antibiotic usage rates, partly due to frequent prescriptions for respiratory and infectious diseases [29]. Specifically, beta‐lactam antibiotics have been identified as the leading cause of drug‐induced anaphylaxis in a population‐based study conducted in Hong Kong [30]. Mislabeling due to inappropriate allergy testing practices, such as pre‐emptive or screening skin tests, historically common in parts of Asia, may also contribute to regional differences in reporting rates [31].

### 4.4 Policy and Clinical Implications

As anaphylaxis requires immediate medical intervention, various severity grading systems, such as the NIAID/FAAN criteria, the World Allergy Organization classification [32] and the Brighton Collaboration Criteria [33], are currently in use. However, these systems rely on subjective assessments, which can be challenging for young children who cannot verbally express their symptoms [34]. Additionally, existing grading systems have limitations in adequately reflecting patients' long‐term outcomes or treatment responses. Thus, establishing a standardised severity grading system, following the recognition and coding of anaphylaxis, can improve diagnostic accuracy and treatment effectiveness.

Notably, the markedly higher reporting rates observed in the Asia‐Pacific region align with an emerging body of literature emphasising regional disparities in drug allergy diagnosis and reporting practices. Recent studies from countries such as South Korea [35], Hong Kong [36] and Vietnam [37] have highlighted both over‐labelling and under‐diagnosis as major challenges. In addition, the negative impact of antibiotic allergy labels, particularly for penicillins, on clinical outcomes, bacterial resistance and healthcare costs has led to growing efforts to remove inaccurate allergy labels [38]. Significantly, skin testing [39] and oral challenges [40] have been effectively implemented for antibiotic allergy delabeling, reinforcing the urgent need for validated allergy testing protocols and increased clinician awareness. Incorporating these perspectives into future guidelines may help address global disparities in allergy management.

### 4.5 Strengths and Limitations

This study provides a comprehensive analysis of the signal detection between all classes of antibiotics and drug‐induced anaphylaxis. However, several limitations should be acknowledged. First, due to the nature of the dataset, which relies on a spontaneous reporting system, some reports may go unreported, potentially leading to over−/underestimation. Given that beta‐lactam antibiotics are among the most widely prescribed antibiotics worldwide [25], their high usage may contribute to a reporting bias in our finding of a significant association with anaphylaxis. Moreover, mislabeling is common, with up to 90% of reported penicillin being inaccurate [41]. This likely leads to overestimation of signals in our dataset, particularly for penicillin. Despite these limitations, the extensive coverage of the database provides valuable insights into global trends and rare adverse events, offering a broader perspective than smaller, localised studies.

Second, this study did not account for variables such as concurrent medication use or pre‐existing medical conditions, both of which could significantly impact the occurrence of adverse events. Although omitting these factors may introduce potential bias, the study's large and diverse dataset, along with appropriate statistical adjustments (ROR and IC), helps mitigate these limitations. Finally, establishing direct causality between antibiotics and anaphylaxis based on our analysis remains challenging due to the observational nature of the data. However, as it is well‐established in previous studies that antibiotics can trigger systemic allergic reactions such as anaphylaxis, our results—showing a high statistical significance—provide strong evidence that all classes of antibiotics are closely associated with anaphylaxis. This supports the importance of careful consideration when prescribing antibiotics to minimise the risk of severe allergic reactions. This approach not only highlights the drugs most strongly detected with anaphylaxis signal but also lays the groundwork for future research and guides improvements in clinical and pharmacovigilance practices.

## 5 Conclusion

While our findings do not permit causal inference, this study is the first to comprehensively analyse the signal detection between all 33 classes of antibiotics and anaphylaxis using the global pharmacovigilance database. Our findings indicate that quinolones and beta‐lactam antibiotics, including penicillins and cephalosporins, accounted for the largest proportion of reports. Thus, efforts to reclassify mislabeled reports with the need for careful history‐taking and diagnostic reassessment are essential.

## Author Contributions

D.K.Y. had full access to all data in the study and took responsibility for the integrity of the data and the accuracy of the data analysis. All authors have approved the final version of the manuscript before submission. Study concept and design: J.C., J.O., J.P. and D.K.Y.; acquisition, analysis, or interpretation of data: J.C., J.O., J.P. and D.K.Y.; drafting of the manuscript: J.C., J.O., J.P. and D.K.Y.; critical revision of the manuscript for important intellectual content: all authors; statistical analysis: J.C., J.O., J.P. and D.K.Y.; study supervision: D.K.Y. D.K.Y. supervised the study and served as the guarantor. J.C., J.O. and J.P. contributed equally as first authors. D.K.Y. and Y.H. contributed equally as corresponding authors. The corresponding author attests that all listed authors meet the authorship criteria, and that no one meeting the criteria has been omitted.

## Ethics Statement

Approval for using confidential and electronically processed patient data was granted by the institutional review board of Kyung Hee University. The requirement for written consent was waived by the ethics committee owing to the population‐level data set.

## Conflicts of Interest

The authors declare no conflicts of interest.

## Supporting information


Data S1.

## Data Availability

Data are available on reasonable request. Study protocol, statistical code: available from DKY (email: yonkkang@gmail.com). Data set: available from the Uppsala Monitoring Centre or World Health Organisation through a data use agreement.
