# Supplementary material for: Global Burden of Drug‐Induced Anaphylaxis Associated With 33 Classes of Antibiotics (1968–2024): A Pharmacovigilance Analysis
Source: Clin Exp Allergy. 2025 Jul 29;55(12):1219–27. doi: 10.1111/cea.70121 (PMC12740533; doi:10.1111/cea.70121)

| **Supplementary Material** |
| --- |

Original Article

**Global burden of drug-induced anaphylaxis associated with 33 classes of antibiotics (1968–2024): A pharmacovigilance analysis**

**Running title:** Antibiotics-associated anaphylaxis

Jaehyeong Cho^1,2#^, Jeongseon Oh^1,3#^, Jaeyu Park^1,3#^, Hyesu Jo^1,4^, Tae Hyeon Kim^1,3^, Hyunjee Kim^1,3^, Yesol Yim^1,3^, Seoyoung Park^1,3^, Kyeongeun Kim^1,3^, Ho Geol Woo^5,6^, Yerin Hwang^1,5^, Michael Miligkos^7^, Dong Keon Yon^1,3,4,5,8*^, Nikolaos G Papadopoulos^7,9^

^#^These authors contributed equally as co-first authors.

***Corresponding author**

**Dong Keon Yon**, MD, PhD, FACAAI, FAAAAI, ATSF

Department of Pediatrics, Kyung Hee University College of Medicine, 23 Kyungheedae-ro, Dongdaemun-gu, Seoul 02447, South Korea

Email: [yonkkang@gmail.com](mailto:yonkkang@gmail.com)

**Table S1.** Medical Dictionary for Regulatory Activities (MedDRA) preferred terms and classifications for anaphylaxis events.

| SOC | HLGT | HLT | PT | LLT | MedDRA code |
| --- | --- | --- | --- | --- | --- |
| Immune system disorders | Allergic conditions | Anaphylactic and anaphylactoid responses | Anaphylactic reaction | Acute anaphyl | 10000662 |
| Immune system disorders | Allergic conditions | Anaphylactic and anaphylactoid responses | Anaphylactic reaction | Acute anaphylactic reaction | 10000663 |
| Immune system disorders | Allergic conditions | Anaphylactic and anaphylactoid responses | Anaphylactic reaction | Acute anaphylaxis | 10000664 |
| Immune system disorders | Allergic conditions | Anaphylactic and anaphylactoid responses | Anaphylactic reaction | Anaphylactic reaction | 10002198 |
| Immune system disorders | Allergic conditions | Anaphylactic and anaphylactoid responses | Anaphylactic reaction | Anaphylactic reaction to chemical | 10054845 |
| Immune system disorders | Allergic conditions | Anaphylactic and anaphylactoid responses | Anaphylactic reaction | Anaphylactic reaction to contrast agent | 10085011 |
| Immune system disorders | Allergic conditions | Anaphylactic and anaphylactoid responses | Anaphylactic reaction | Anaphylactic reaction to drug | 10054844 |
| Immune system disorders | Allergic conditions | Anaphylactic and anaphylactoid responses | Anaphylactic reaction | Anaphylactic reaction to food | 10054843 |
| Immune system disorders | Allergic conditions | Anaphylactic and anaphylactoid responses | Anaphylactic reaction | Anaphylactic reaction to vaccine | 10063979 |
| Immune system disorders | Allergic conditions | Anaphylactic and anaphylactoid responses | Anaphylactic reaction | Anaphylactic reaction to venom | 10073013 |
| Immune system disorders | Allergic conditions | Anaphylactic and anaphylactoid responses | Anaphylactic reaction | Anaphylaxis | 10002218 |
| Immune system disorders | Allergic conditions | Anaphylactic and anaphylactoid responses | Anaphylactic reaction | Biphasic anaphylactic reaction | 10086843 |
| Immune system disorders | Allergic conditions | Anaphylactic and anaphylactoid responses | Anaphylactic reaction | Exercise-induced anaphylaxis | 10060689 |
| Immune system disorders | Allergic conditions | Anaphylactic and anaphylactoid responses | Anaphylactic reaction | Reaction anaphylactic anaphylactoid | 10037933 |
| Immune system disorders | Allergic conditions | Anaphylactic and anaphylactoid responses | Anaphylactic reaction | Systemic anaphylactic reaction | 10042930 |
| Immune system disorders | Allergic conditions | Anaphylactic and anaphylactoid responses | Anaphylactic reaction | Systemic anaphylaxis | 10042931 |
| Immune system disorders | Allergic conditions | Anaphylactic and anaphylactoid responses | Anaphylactic shock | Allergic shock | 10069526 |
| Immune system disorders | Allergic conditions | Anaphylactic and anaphylactoid responses | Anaphylactic shock | Anaphylactic shock | 10002199 |
| Immune system disorders | Allergic conditions | Anaphylactic and anaphylactoid responses | Anaphylactic shock | Anaphylactic shock due to adverse food reaction | 10002200 |
| Immune system disorders | Allergic conditions | Anaphylactic and anaphylactoid responses | Anaphylactic shock | Anaphylactic shock due to crustaceans | 10002201 |
| Immune system disorders | Allergic conditions | Anaphylactic and anaphylactoid responses | Anaphylactic shock | Anaphylactic shock due to eggs | 10002202 |
| Immune system disorders | Allergic conditions | Anaphylactic and anaphylactoid responses | Anaphylactic shock | Anaphylactic shock due to fish | 10002203 |
| Immune system disorders | Allergic conditions | Anaphylactic and anaphylactoid responses | Anaphylactic shock | Anaphylactic shock due to food additives | 10002204 |
| Immune system disorders | Allergic conditions | Anaphylactic and anaphylactoid responses | Anaphylactic shock | Anaphylactic shock due to fruits and vegetables | 10002205 |
| Immune system disorders | Allergic conditions | Anaphylactic and anaphylactoid responses | Anaphylactic shock | Anaphylactic shock due to milk products | 10002206 |
| Immune system disorders | Allergic conditions | Anaphylactic and anaphylactoid responses | Anaphylactic shock | Anaphylactic shock due to other specified food | 10002207 |
| Immune system disorders | Allergic conditions | Anaphylactic and anaphylactoid responses | Anaphylactic shock | Anaphylactic shock due to peanuts | 10002208 |
| Immune system disorders | Allergic conditions | Anaphylactic and anaphylactoid responses | Anaphylactic shock | Anaphylactic shock due to tree nuts and seeds | 10002210 |
| Immune system disorders | Allergic conditions | Anaphylactic and anaphylactoid responses | Anaphylactic shock | Anaphylactic shock due to unspecified food | 10002211 |
| Immune system disorders | Allergic conditions | Anaphylactic and anaphylactoid responses | Anaphylactic shock | Anaphylactic shock, not elsewhere classified | 10002212 |
| Immune system disorders | Allergic conditions | Anaphylactic and anaphylactoid responses | Anaphylactic shock | Drug shock | 10013743 |
| Immune system disorders | Allergic conditions | Anaphylactic and anaphylactoid responses | Anaphylactic shock | Penicillin shock | 10034293 |
| Immune system disorders | Allergic conditions | Anaphylactic and anaphylactoid responses | Anaphylactic shock | Shock anaphylactic anaphylactoid | 10040562 |
| Immune system disorders | Allergic conditions | Anaphylactic and anaphylactoid responses | Anaphylactoid reaction | Anaphylactic type reaction | 10002213 |
| Immune system disorders | Allergic conditions | Anaphylactic and anaphylactoid responses | Anaphylactoid reaction | Anaphylactoid reaction | 10002216 |
| Immune system disorders | Allergic conditions | Anaphylactic and anaphylactoid responses | Anaphylactoid reaction | Complement activation-related pseudoallergy | 10088687 |
| Immune system disorders | Allergic conditions | Anaphylactic and anaphylactoid responses | Anaphylactoid reaction | Delayed anaphylactoid reaction | 10057789 |
| Immune system disorders | Allergic conditions | Anaphylactic and anaphylactoid responses | Anaphylactoid reaction | Pseudoallergic reaction | 10084859 |
| Immune system disorders | Allergic conditions | Anaphylactic and anaphylactoid responses | Anaphylactoid shock | Anaphylactoid shock | 10063119 |

 Abbreviations: HLT, high-level term; HLGT, high-level group term; LLT, lower-level terms; PT, preferred terms; SOC, system organ class.

**Table S2.** Subgroup analysis of individuals drug-induced anaphylaxis disparities.

|  | Total | drug-induced anaphylaxis | | | IC (IC_0.25_) based on age, years | | | | |
| --- | --- | --- | --- | --- | --- | --- | --- | --- | --- |
|  |  | Observed | ROR (95% CI) | IC (IC_0.25_) | 1-17 | 18-44 | 44-64 | 65-74 | ≥75 |
| **Total** | 3,328,572 | 144,820 | **20.50 (20.37 - 20.63)** | **3.77 (3.76)** | **3.06 (3.03)** | **3.37 (3.35)** | **3.77 (3.76)** | **3.80 (3.78)** | **3.61 (3.58)** |
| **Sex difference** |  |  |  |  |  |  |  |  |  |
| Male | 1,426,070 | 64,823 | **19.70 (19.51 - 19.90)** | **3.65 (3.64)** | **3.04 (3.00)** | **3.26 (3.24)** | **3.55 (3.53)** | **3.58 (3.55)** | **3.53 (3.49)** |
| Female | 1,780,689 | 78,144 | **20.93 (20.75 - 21.11)** | **3.84 (3.83)** | **3.07 (3.02)** | **3.43 (3.41)** | **3.92 (3.90)** | **3.98 (3.95)** | **3.67 (3.64)** |
| **Antibiotics class** |  |  |  |  |  |  |  |  |  |
| Tetracyclines | 103,684 | 1,060 | **3.25 (3.06 - 3.45)** | **1.68 (1.58)** | **1.34 (0.99)** | **1.34 (1.18)** | **1.54 (1.34)** | **1.72 (1.42)** | **0.85 (0.34)** |
| Amphenicols | 8,107 | 123 | **4.84 (4.05 - 5.78)** | **2.23 (1.94)** | 0.70 (-0.38) | **2.06 (1.60)** | **2.31 (1.79)** | **2.08 (1.21)** | 1.02 (-0.74) |
| Beta-lactam antibacterials, penicillins | 761,055 | 39,696 | **18.82 (18.62 - 19.01)** | **4.04 (4.02)** | **2.60 (2.54)** | **3.57 (3.54)** | **4.17 (4.14)** | **4.23 (4.19)** | **4.09 (4.04)** |
| Penicillins with extended spectrum | 256,126 | 13,348 | **17.74 (17.43 - 18.06)** | **4.04 (4.01)** | **2.11 (2.01)** | **3.55 (3.50)** | **4.34 (4.29)** | **4.47 (4.40)** | **4.22 (4.12)** |
| Beta-lactamase sensitive penicillins | 89,770 | 8,099 | **31.66 (30.94 - 32.40)** | **4.83 (4.79)** | **3.26 (3.14)** | **4.40 (4.34)** | **4.97 (4.91)** | **5.04 (4.94)** | **5.20 (5.07)** |
| Beta-lactamase resistant penicillins | 67,653 | 2,017 | **9.68 (9.26 - 10.12)** | **3.23 (3.15)** | **2.23 (2.03)** | **2.90 (2.77)** | **3.27 (3.14)** | **3.13 (2.91)** | **2.71 (2.43)** |
| Beta-lactamase inhibitors | 2,855 | 103 | **11.75 (9.65 - 14.30)** | **3.43 (3.11)** | **2.24 (0.93)** | **3.27 (2.64)** | **3.38 (2.83)** | **2.82 (1.95)** | **2.40 (1.33)** |
| Combinations of penicillins, incl. beta-lactamase inhibitors | 344,651 | 16,129 | **15.93 (15.68 - 16.19)** | **3.88 (3.85)** | **2.83 (2.75)** | **3.28 (3.23)** | **3.90 (3.86)** | **4.01 (3.95)** | **3.96 (3.89)** |
| Other beta-lactam antibacterials | 899,788 | 63,644 | **27.59 (27.35 - 27.83)** | **4.48 (4.46)** | **3.83 (3.79)** | **4.05 (4.03)** | **4.41 (4.38)** | **4.48 (4.45)** | **4.31 (4.27)** |
| First-generation cephalosporins | 138,001 | 9,294 | **23.11 (22.62 - 23.60)** | **4.40 (4.37)** | **3.61 (3.50)** | **4.04 (3.98)** | **4.22 (4.15)** | **4.37 (4.27)** | **4.12 (3.99)** |
| Second-generation cephalosporins | 185,225 | 14,484 | **27.46 (26.99 - 27.93)** | **4.62 (4.59)** | **3.70 (3.63)** | **4.18 (4.13)** | **4.63 (4.58)** | **4.67 (4.59)** | **4.59 (4.49)** |
| Third-generation cephalosporins | 459,516 | 38,083 | **30.84 (30.51 - 31.18)** | **4.70 (4.69)** | **4.03 (3.99)** | **4.20 (4.17)** | **4.61 (4.58)** | **4.75 (4.71)** | **4.64 (4.59)** |
| Fourth-generation cephalosporins | 19,804 | 364 | **5.88 (5.30 - 6.52)** | **2.52 (2.35)** | **2.14 (1.61)** | **2.36 (1.99)** | **2.30 (1.98)** | **2.39 (1.98)** | **1.94 (1.44)** |
| Monobactams | 23,050 | 644 | **9.03 (8.35 - 9.77)** | **3.13 (3.00)** | **2.37 (1.99)** | **2.65 (2.38)** | **3.65 (3.43)** | **3.32 (3.02)** | **3.11 (2.80)** |
| Carbapenems | 70,713 | 737 | **3.31 (3.08 - 3.56)** | **1.71 (1.17)** | **1.65 (1.27)** | **1.59 (1.32)** | **1.61 (1.40)** | **1.41 (1.11)** | **1.37 (1.06)** |
| Other cephalosporins and penems | 3,479 | 38 | **3.47 (2.52 - 4.77)** | **1.02 (0.82)** | N/A | **2.24 (1.92)** | **1.80 (1.41)** | **1.25 (0.59)** | 0.44 (-0.10) |
| Sulfonamides and trimethoprim | 175,097 | 1,849 | **3.36 (3.21 - 3.52)** | **1.73 (1.66)** | **0.91 (0.66)** | **1.45 (1.33)** | **1.64 (1.49)** | **1.32 (1.07)** | **1.09 (0.79)** |
| Trimethoprim and derivatives | 12,871 | 263 | **6.55 (5.80 - 7.40)** | **2.67 (2.47)** | 1.37 (-0.70) | **2.74 (0.15)** | **2.56 (1.26)** | **1.91 (0.15)** | 2.17 (-0.42) |
| Short-acting sulfonamides | 2,031 | 23 | **3.59 (2.38 - 5.42)** | **0.73 (0.25)** | 1.17 (-0.90) | -0.24 (-2.84) | **1.68 (0.38)** | 1.58 (-0.18) | 0.89 (-1.70) |
| Intermediate-acting sulfonamides | 5,071 | 49 | **3.06 (2.31 - 4.06)** | **1.58 (0.55)** | -0.19 (-2.79) | **1.23 (0.50)** | **1.11 (0.09)** | **1.93 (0.63)** | 0.05 (-3.73) |
| Long-acting sulfonamides | 3,103 | 11 | **1.12 (0.62 - 2.02)** | **8.52 (8.43)** | -2.05 (-2.31) | **0.80 (0.66)** | **0.58 (0.42)** | 0.12 (-0.17) | N/A |
| Combinations of sulfonamides and trimethoprim, incl. derivatives | 152,021 | 1,503 | **3.14 (2.98 - 3.30)** | **1.64 (1.55)** | **0.98 (0.87)** | **1.32 (1.22)** | **1.54 (1.43)** | **1.18 (1.02)** | **0.79 (0.55)** |
| Macrolides, lincosamides and streptogramins | 335,137 | 8,455 | **8.26 (8.08 - 8.44)** | **2.99 (2.95)** | **2.52 (2.42)** | **2.71 (2.64)** | **2.97 (2.91)** | **2.92 (2.81)** | **2.57 (2.42)** |
| Streptogramins | 229,225 | 3,798 | **5.32 (5.15 - 5.50)** | **2.38 (2.33)** | **2.21 (2.10)** | **1.99 (1.90)** | **2.25 (2.15)** | **2.30 (2.14)** | **1.89 (1.66)** |
| Lincosamides | 99,351 | 4,415 | **14.73 (14.29 - 15.18)** | **3.80 (3.75)** | **3.50 (3.33)** | **3.53 (3.45)** | **3.67 (3.58)** | **3.63 (3.49)** | **3.45 (3.26)** |
| Macrolides | 6,561 | 242 | **12.02 (10.57 - 13.67)** | **3.51 (3.26)** | 0.41 (-3.37) | **3.60 (3.23)** | **3.64 (3.31)** | **2.65 (1.98)** | 0.36 (-1.06) |
| Aminoglycoside antibacterials | 92,558 | 1,919 | **6.67 (6.37 - 6.98)** | **2.70 (2.63)** | **1.65 (1.37)** | **2.47 (2.33)** | **2.95 (2.82)** | **2.80 (2.59)** | **2.60 (2.35)** |
| Streptomycins | 6,239 | 185 | **9.59 (8.29 - 11.10)** | **3.19 (2.95)** | **2.76 (1.78)** | **2.54 (2.14)** | **3.33 (2.95)** | **2.62 (1.81)** | 1.36 (-0.41) |
| Other aminoglycosides | 86,319 | 1,734 | **6.45 (6.15 - 6.77)** | **2.66 (2.64)** | **1.56 (1.27)** | **2.45 (2.31)** | **2.89 (2.75)** | **2.79 (2.58)** | **2.64 (2.38)** |
| Quinolone antibacterials | 515,980 | 20,303 | **13.40 (13.21 - 13.60)** | **3.63 (3.61)** | **2.83 (2.66)** | **3.44 (3.40)** | **3.58 (3.54)** | **3.47 (3.41)** | **3.10 (3.03)** |
| Fluoroquinolones | 511,183 | 20,130 | **13.41 (13.22 - 13.60)** | **3.63 (3.61)** | **2.86 (2.68)** | **3.44 (3.40)** | **3.58 (3.54)** | **3.46 (3.41)** | **3.10 (3.03)** |
| Other quinolones | 4,797 | 173 | **11.74 (10.09 - 13.67)** | **3.46 (3.16)** | 1.30 (-0.26) | **2.84 (2.40)** | **3.36 (2.92)** | **3.38 (2.74)** | **2.89 (2.02)** |
| Combinations of antibacterials | 8,586 | 122 | **4.52 (3.78 - 5.41)** | **2.14 (1.84)** | **1.64 (0.67)** | **1.65 (1.20)** | **2.23 (1.72)** | 1.19 (-0.22) | -0.08 (-3.86) |
| Other antibacterials | 428,580 | 7,649 | **5.78 (5.65 - 5.92)** | **2.49 (2.45)** | **2.02 (1.89)** | **2.26 (2.20)** | **2.35 (2.28)** | **2.25 (2.14)** | **1.98 (1.85)** |
| Glycopeptide antibacterials | 122,875 | 2,470 | **6.47 (6.21 - 6.73)** | **2.66 (2.27)** | **2.15 (1.97)** | **2.35 (2.21)** | **2.39 (2.26)** | **2.38 (2.20)** | **2.72 (2.53)** |
| Polymyxins | 12,639 | 73 | **1.82 (1.45 - 2.29)** | **0.85 (0.47)** | 0.92 (-0.49) | **1.20 (0.54)** | 0.29 (-0.56) | 0.39 (-0.64) | 0.00 (-1.30) |
| Steroid antibacterials | 5,305 | 39 | **2.32 (1.70 - 3.18)** | **1.19 (1.13)** | **1.62 (0.72)** | 0.56 (-0.74) | **1.09 (0.01)** | 0.58 (-1.19) | 0.20 (-1.87) |
| Imidazole derivatives | 126,011 | 3,218 | **8.27 (7.99 - 8.57)** | **3.01 (2.95)** | **1.77 (1.44)** | **2.66 (2.57)** | **2.90 (2.79)** | **2.96 (2.79)** | **2.44 (2.19)** |
| Nitrofuran derivatives | 32,113 | 279 | **2.75 (2.45 - 3.10)** | **1.45 (1.36)** | -1.58 (-5.36) | **0.92 (0.47)** | **1.48 (1.13)** | **1.24 (0.76)** | **0.78 (0.21)** |
| Other antibacterials | 129,637 | 1,570 | **3.86 (3.67 - 4.05)** | **1.93 (1.85)** | **2.16 (1.87)** | **1.70 (1.56)** | **1.85 (1.71)** | **1.75 (1.54)** | **0.99 (0.66)** |

Abbreviation: IC; information component, ROR; reporting odds ratio.

Bold style indicates when the value of IC_0.25_ is greater than 0.00 or the lower end of the ROR 95% CI is greater than 1.00. This means it is statistically significant.

**Figure S1.** Representation of the cumulative number of reports of drug-induced anaphylaxis events by year, categorized by 10 classes of antibiotics.


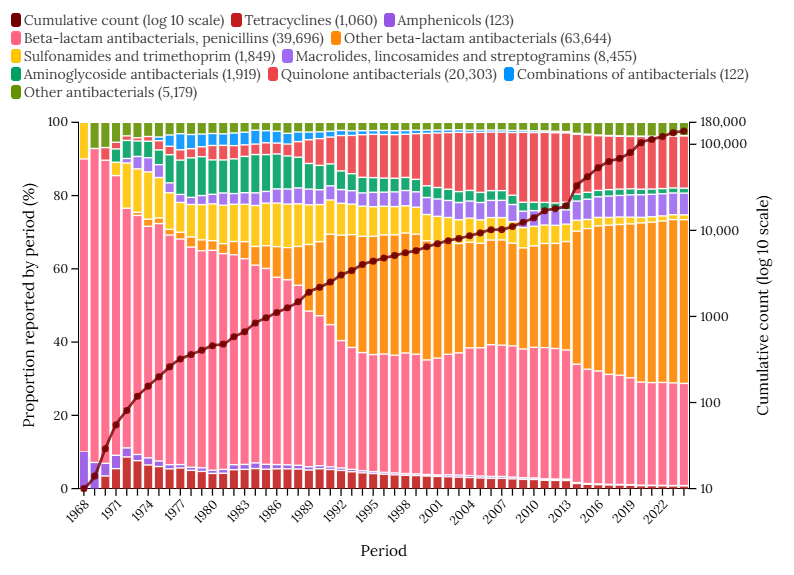

Supplement: Supplementary file 1 — Data S1. [file CEA-55-1219-s001.docx]
